# Supplementary material for: Content-rich biological network constructed by mining PubMed abstracts
Source: BMC Bioinformatics. 2004 Oct 8;5:147. doi: 10.1186/1471-2105-5-147 (PMC528731; doi:10.1186/1471-2105-5-147)
Supplement: Additional File 2 — The original results of the above study (non-essential files are deleted to keep the file size under the limit set by BMC bioinformatics). [file 1471-2105-5-147-S2.bz2 › chilibotAdditionalFile2/dip20/17ID9194558E65/html/BAX_BCL2.html]

 


 **BAX** and **BCL2** 
  
Found 3169 abstracts in PubMed, retrieved 20.  
 

 What does Google say? 
 PDF only 
| .edu only 

---

**Interactive relationship** (e.g. stimulation, inhibition, etc)

**Inhibitory relationship**- Expression of the anti apoptosis proteins, Bcl 2  [ **BCL2** ]  and Mcl 1 expression decreased time dependently while  **Bax**  expression increased.  Ref: 12883267 Pancreas, 2003
**Neutral relationship**- Furthermore, it down regulated the expression of Bcl 2  [ **BCL2** ]  and poly ADP ribose polymerase, but up regulated the expression of caspase 3 and  **Bax**  in dopaminergic SK N SH neurons.  Ref: 12880480 Antioxid Redox Signal, 2003

**Non-interactive relationship** (e.g. studied together, co-existance, homology, etc.)

- CONCLUSIONS AGE can induce a significant apoptosis of BRPs in a dose dependent manner and the rate of apoptosis was determined by the  **Bax**  bcl 2  [ **BCL2** ]  ratio.  Ref: 12882701 Zhonghua Yan Ke Za Zhi, 2003
- Thus, EGCG had a concurrent effect on two important transcription factors p53 and NF kappaB, causing a change in the ratio of  **Bax**  Bcl 2  [ **BCL2** ]  in a manner that favors apoptosis.  Ref: 12894226 Oncogene, 2003
- STUDY DESIGN Tissue sections from 60 NSCLCs were assessed for the expression of p53, Ki 67, bcl 2  [ **BCL2** ] ,  **bax**  and FasL using immunohistochemistry and antibodies.  Ref: 12882088 Anal Quant Cytol Histol, 2003
- treatment of melanocytes with either 5 OH 2 D 3 or S1P did not alter the Bcl 2  [ **BCL2** ]   **Bax**  ratio.  Ref: 12883359 Melanoma Res, 2003
- The present results suggest that Phx demonstrates antitumor activity against human endometrial adenocarcinoma cell lines EN and KLE cells, by inducing both cell cycle accumulation at S and G2M and apoptosis associated with p53, Bcl 2  [ **BCL2** ]  and  **Bax**  insensitive pathways.  Ref: 12883676 Oncol RepOncol Rep, 2003
- Here, we show that both proapoptotic Bcl 2  [ **BCL2** ]  family members Bak and  **Bax**  were activated by IFNalpha, strictly in correlation with the induction of apoptosis.  Ref: 12881711 Oncogene, 2003
- Changes in the expression of caspase 3,  **bax** , bcl 2  [ **BCL2** ]  and p21cip waf were assessed by quantitative Western blotting.  Ref: 12888913 Int J Oncol, 2003
- To examine what factors can precisely predict the radio sensitivity or radio resistance of early stage laryngeal squamous cell carcinomas LSCC, protein expression of p53, Bcl 2  [ **BCL2** ]  and  **Bax**  and loss of heterozygosity LOH at 3p and 9p loci were investigated.  Ref: 12883724 Oncol RepOncol Rep, 2003
- However, when cultured, lymphocytes treated with ECP showed a rapid downregulation of Bcl 2  [ **BCL2** ] , an upregulation of  **bax**  with an increased  **bax**  Bcl 2  [ **BCL2** ]  ratio, a decrease in bcl 2  [ **BCL2** ]  mRNA and an increase in Fas.  Ref: 12883827 Arch Dermatol ResArch Dermatol Res, 2003
- The level of Bcl 2  [ **BCL2** ]  remained largely unchanged, but the  **Bax** , Fas, and FasL expression showed up regulation.  Ref: 12880947 Biochim Biophys Acta, 2003
- Interferon alpha induced apoptosis in U266 cells is associated with activation of the proapoptotic Bcl 2  [ **BCL2** ]  family members Bak and  **Bax** .  Ref: 12881711 Oncogene, 2003
- In addition, up regulation of  **Bax**  and down regulation of Bcl 2  [ **BCL2** ]  was also observed indicating that these two apoptotic regulators may play important roles in terazosin mediated cell death pathway.  Ref: 12883741 Oncol RepOncol Rep, 2003
- The purpose of this study was to characterize the roles in retinal development of the proapoptotic Bcl 2  [ **BCL2** ]  family members  **Bax**  and Bak.  Ref: 12882813 Invest Ophthalmol Vis SciInvest Ophthalmol Vis Sci, 2003
- CONCLUSION survivin gene may play an important role in pathogenesis of ovarian cancer, apoptosis related gene bcl 2  [ **BCL2** ]  may have a synergic role and  **bax**  have an antagonistic role with survivin gene in formation and progression of ovarian cancer.  Ref: 12885364 Zhonghua Fu Chan Ke Za Zhi, 2003
- Expression and function of apoptosis related genes Bcl 2  [ **BCL2** ]   **Bax**  and Fas Fas L in the course of stress ulcer .  Ref: 12887767 Zhonghua Yi Xue Za Zhi, 2003
- ZD1839 alone induced accumulation of cells in the G0 G1 phase of the cell cycle at 24 h accompanied by a concomitant increase in p21, p27 and  **Bax** , a significant decrease in  **Bcl2**  and a decrease in Akt phosphorylation.  Ref: 12888834 Br J Cancer, 2003
- The expression of Bcl 2  [ **BCL2** ]   **Bax**  protein was examined by immunohistochemical assay.  Ref: 12887767 Zhonghua Yi Xue Za Zhi, 2003
- Immediately after isolation, there was no difference in Bcl 2  [ **BCL2** ]  or  **bax**  expression among the four different samples, or in Fas and FasL mRNA.  Ref: 12883827 Arch Dermatol ResArch Dermatol Res, 2003
- RT PCR demonstrated that the pro apoptic gene  **bax**  was induced while the anti apoptic gene bcl 2  [ **BCL2** ]  and cell cycle active gene PCNA were reduced.  Ref: 12883747 Oncol RepOncol Rep, 2003
- As both 5 OH 2 D 3 and S1P have been identified to modify the Bcl 2  [ **BCL2** ]   **Bax**  ratio in epithelial cells, we also measured the expressions of these proteins.  Ref: 12883359 Melanoma Res, 2003
- Proapoptotic Bcl 2  [ **BCL2** ]  Family Members,  **Bax**  and Bak, Are Essential for Developmental Photoreceptor Apoptosis.  Ref: 12882813 Invest Ophthalmol Vis SciInvest Ophthalmol Vis Sci, 2003
- METHODS With the methods of in situ hybridization and immunohistochemistry staining, PKC and apoptosis gene Bcl 2  [ **BCL2** ] ,  **Bax**  expression were measured between nasal polyps from 26 patients and inferior turbinate mucous membrane tissue ITMMT from 20 normal persons.  Ref: 12889099 Zhonghua Er Bi Yan Hou Ke Za Zhi, 2003
- Apoptosis of these cells caused by Phx was unlikely to be associated with p53,  **Bax** , and Bcl 2  [ **BCL2** ] , because the levels of these proteins were not altered regardless of the presence or absence of Phx.  Ref: 12883676 Oncol RepOncol Rep, 2003
- OBJECTIVE To study the expression of apoptosis related gene survivin and its relation with expression of bcl 2  [ **BCL2** ] ,  **bax**  protein in epithelial ovarian cancer.  Ref: 12885364 Zhonghua Fu Chan Ke Za Zhi, 2003
- METHODS After a 4 day incubation with various concentrations of AGE 2 0 000 mg L which were prepared in vitro by incubating bovine serum albumin BSA with glucose, we studied the degree of apoptosis and the expressions of apoptotic genes  **Bax**  and bcl 2  [ **BCL2** ]  in BRPs by staining with the Annexin V FITC and propidium iodide PI, terminal deoxynucleotidyl transferase mediated dUTP nick end labeling TUNEL and immunohistochemistry.  Ref: 12882701 Zhonghua Yan Ke Za Zhi, 2003
- Expression of bcl 2  [ **BCL2** ]  and  **bax**  protein was positively and negatively correlated with expression of survivin gene respectively.  Ref: 12885364 Zhonghua Fu Chan Ke Za Zhi, 2003
- The combination effectively up regulated caspase 3 and  **bax**  and down regulated bcl 2  [ **BCL2** ]  and p21cip waf.  Ref: 12888913 Int J Oncol, 2003
- To elucidate molecular events in the apoptosis, protein expressions of Bcl 2  [ **BCL2** ] ,  **Bax** , Fas, and FasL were measured by Western blotting using specific antibodies in HL 60 cells.  Ref: 12880947 Biochim Biophys Acta, 2003
- Expression of bcl 2  [ **BCL2** ]  and  **bax**  was detected by immunohistochemistry streptomycin avidin biotin peroxidase complex SABC method, and correlation between them was analyzed.  Ref: 12885364 Zhonghua Fu Chan Ke Za Zhi, 2003
- Expression of survivin gene and its relation with the expression of bcl 2  [ **BCL2** ]  and  **bax**  protein in epithelial ovarian cancer .  Ref: 12885364 Zhonghua Fu Chan Ke Za Zhi, 2003
- There was no difference found in the expression of proapoptotic  **Bax**  and Bcl X s or antiapoptotic Bcl 2  [ **BCL2** ]  and Bcl X L genes nor in the expression of the tumor suppressor gene p53.  Ref: 12885947 J Leukoc Biol, 2003
- Bcl 2  [ **BCL2** ]  and  **bax**  quantified immunostaining demonstrated a weak negative correlation with shape factor values P =.039 and P =.025, respectively.  Ref: 12882088 Anal Quant Cytol Histol, 2003
- OBJECTIVE To investigate the correlation between the expressions of apoptosis promoting gene  **Bax** , apotosis inhibiting gene Bcl 2  [ **BCL2** ] , and Fas FasL in gastric mucosa in the course of stress ulcer.  Ref: 12887767 Zhonghua Yi Xue Za Zhi, 2003
- Overexpression of Bcl 2  [ **BCL2** ]  blocked apoptosis induced by IFNalpha totally abolished Bak activation, as well as decreased the amount of activated  **Bax** .  Ref: 12881711 Oncogene, 2003
- 4 There was a positive correlation between apoptotic rate and  **Bax**  bcl 2  [ **BCL2** ]  ratio of BRPs induced by AGE r = 0.808, P <.  Ref: 12882701 Zhonghua Yan Ke Za Zhi, 2003
